# Clinical Effects of the ‘Crisis Toolbox’ (CTB): A Brief, Skills Based, Intervention Delivered in a Crisis Resolution and Home Treatment Team

**DOI:** 10.1007/s10597-023-01100-6

**Published:** 2023-03-26

**Authors:** Lee D. Mulligan, Sandra T. Neil, Lesley-Anne Carter, Georgia DeBank, Megan Johnstone, Katie Fox, Dominic Veakins

**Affiliations:** 1 Division of Biology, Medicine & Health, School of Health Sciences, University of Manchester, Oxford Road, M13 9PL Manchester, UK; 2 Atherleigh Park Hospital, Greater Manchester Mental Health NHS Foundation Trust, Atherleigh Way, WN7 1YN Leigh, UK; 3 Centre for Biostatistics, University of Manchester, Oxford Road, M13 9PL Manchester, UK

**Keywords:** Adult mental health, Crisis resolution home treatment, Brief psychological interventions, Telephone interventions, Service evaluation.

## Abstract

Access to psychological interventions for people under Crisis Resolution and Home Treatment Teams (CRHTTs) is limited. The Crisis Toolbox (CTB) is a skills-based intervention designed to increase access using flexible methods of delivery. This study aimed to evaluate the clinical effects of the CTB. A retrospective service evaluation of 399 participants who accessed the CTB between November 2020 and February 2021 was employed. Sessional measures comprising the Patient Health Questionnaire (PHQ-9) and Generalised Anxiety Scale (GAD-7) were recorded across three time points. Overall, there were significantly decreasing trends in PHQ-9 (β = − 1.6, *p* < 0.001) and GAD-7 scores (β = − 1.5, *p* < 0.001) in participants who accessed the CTB. The magnitude and direction of specific trends differed according to age, diagnosis, and neurodiversity. The CTB could help reduce depression and anxiety in people experiencing crisis. Randomised controlled trials are now required to test its acceptability, feasibility, and effectiveness.

## Introduction

The last twenty years has seen significant National Health Service (NHS) transformation for people experiencing mental health crisis. Since the publication of the National Service Framework (NSF) for Mental Health [Department of Health (DoH), 1999], the NHS Plan (DoH, 2000) and Mental Health Policy Implementation Guide (MHPIG) (DoH, 2001), there has been significant importance placed on the development of accessible, community-based, acute care services. Consequently, Crisis Resolution Home Treatment Teams (CRHTTs) were developed and remain a financial priority (NHS England, 2019).

CRHTTs aim to deliver 24 h, 7 days per week mental health services for people experiencing severe and enduring mental health problems, and whom, without crisis intervention, would likely require hospitalisation (DoH, 2001). CRHTTs aim to support people in a least restrictive environment through the provision of immediate multidisciplinary care and flexible, time-limited intervention. Research evidence from both randomised controlled trials (RCTs) and non-randomised studies have demonstrated that CRHTTs can reduce both hospital admissions (Johnson et al., 2005) and length of inpatient stays (Stulz et al., 2020). However, problems with the CRHTT model have also been identified and not all findings have been positive (Tyrer et al., 2010; Jacobs & Barrenho, 2011). Reasons for heterogeneity in outcomes could be related to several factors, including the availability of staffing and resources, establishment of referral pathways, effectiveness of multidisciplinary working, and accessibility of staff training and development (Crompton & Daniel, 2007).

Clinical psychologists can play several key roles in CRHTTs [British Psychological Society (BPS), 2008]. In terms of direct provision, they can provide a repertoire of brief, evidence-based, therapies, with proven efficacy for those presenting with self-harm and/or suicidal thoughts or behaviours [Royal College of Psychiatrists (RCP), 2019]. These include Cognitive Behavioural Therapy (CBT; Beck, Rush, Shaw & Emery, 1979) (Tarrier et al., 2008), Dialectical Behavioural Therapy (DBT; Linehan, 2018) and Eye Movement Desensitization and Reprocessing (EMDR: Shapiro, 2017) (Proudlock & Peris, 2020). Despite this, access to psychological interventions within CRHTT settings is problematic [Ebrahim, 2021; Association of Clinical Psychology UK (ACP-UK), 2021] and where specific interventions have been implemented, many require specialist training to deliver. This has encouraged the development of brief, skills-based interventions, in which clinical psychologists play an indirect role through provision of training and supervision (BPS, 2008).

Brief, skills-based, interventions have proven efficacy in the treatment of suicidal thoughts and / or behaviours across various urgent care settings (Yardley et al., 2019; Guthrie et al., 2001; McCabe, Garside, Backhouse, Xanthopoulou, 2018; Chopra et al., 2021). However, it is unclear if these positive findings generalise to CRHTTs, which have different operational challenges and support populations with unique and diverse intersectional needs. Furthermore, the impact of Coronavirus disease 2019 (COVID-19) not only increased demand on mental health services but encouraged them to transform and offer more services remotely, for example, via telephone (Zhou et al., 2020). Whilst there is a well-established evidence base for the delivery of telephonic psychological interventions in primary care (Simon et al., 2004), there is a relative lack of research on the efficacy of telephonic interventions for those accessing urgent care. Indeed, only one study in Australia found that brief interventions delivered via telephone led to significant decreases in distress for those presenting in crisis (Bidargaddi et al., 2015). Therefore, there is an outstanding need for UK CRHTTs to develop brief interventions that are amenable to both face-to-face and telephonic delivery and relevant to the populations they serve.

One CRHTT in Greater Manchester recently developed a brief, skills based, intervention to fill this gap in service provision. The ‘Crisis Toolbox’ (CTB) was adapted from an existing intervention utilised across CRHTTs in the North-West of England in response to the COVID-19 pandemic. The CTB comprised nine skills pertinent to crises management, each drawn from existing evidence-based psychological interventions with promising effects on self-harm and suicide (Calati & Courtet, 2016; Witt et al., 2021). This included distress tolerance, problem solving, the STOPP skill, distraction techniques, self-soothing (Linehan, 2018), surf the urge (Marlatt & Donovon, 2005), sleep hygiene (Hauri, 1991), grounding (Lowen, 1958) and worry management (Butler & Hope, 2007). The implementation of these skills was manualised, formulation driven (i.e., selected based on the needs of each individual service user) and flexible (i.e., delivered either face-to-face or via telephone), and were used to supplement crisis safety planning delivered by members of the wider CRHTT. The CTB has been previously shown to have high acceptability for those experiencing mental health crises (Mulligan et al., 2022). However, it remains unknown if the CTB has a positive effect on psychological distress.

This project aimed to explore this gap and retrospectively evaluate the clinical effects of the CTB on routinely collected outcome measures.

## Methods

### Design

A retrospective service evaluation of routinely collected demographic, clinical and outcome data was employed to evaluate the clinical effects of the CTB. These outcome measures were collected as part of routine provision of the CTB and not the CRHTT service as a whole.

### Intervention

The CTB comprised up to three sessions of brief, psychologically informed skills and was offered to all individuals referred under the CRHTT. Its aim was to provide service-users with a range of coping skills and strategies to utilise when experiencing crises. Specific modules included: (1) distress tolerance; (2) problem solving; (3) the STOPP skill; (4) surf the urge; (5) distraction; (6) sleep hygiene; (7) self-soothing; (8) grounding; and (9) worry management. These were chosen given their relevance to crisis management. To maximise consistency in delivery, the CTB was manualised and afforded practitioners the flexibility to introduce different skills based on an individual’s presenting needs. All CTB sessions were conducted via telephone or face-to-face by Assistant Psychologists (APs), or a Trainee Associate Psychological Practitioner (TAPP), under the supervision of a qualified Clinical Psychologist.

### Measures

For each participant accessing the CTB, demographic (i.e., age, gender) and clinical (i.e., primary / secondary diagnosis) measures were recorded by practitioners via scrutiny of medical records. Two outcome measures were also recorded at the start of each CTB session (i.e., time 1, time 2 and time 3). Both measures were administered via telephone or face-to-face depending on the delivery of the CTB.

#### The Patient Health Questionnaire (PHQ-9)

The nine-item PHQ-9 (Kroenke et al., 2001) is a composite measure of depression. All items are rated on ordinal scales (0 = Not at all, 4 = Nearly every day) and total scores range from 0 to 36. The PHQ-9 has been widely used as a routine outcome measure in mental health services and has demonstrable reliability and validity (Cameron et al., 2008).

#### Generalised Anxiety Disorder Assessment (GAD-7)

The seven-item GAD-7 (Spitzer, Kroenke, Williams & Lowe, 2006) is composite measure of anxiety. All items are rated on ordinal scales (0 = Not at all, 4 = Nearly every day) and total scores range from 0 to 28. The GAD-7 has been widely used as a routine outcome measure for mental health services and has demonstrable reliability and validity in heterogenous samples (Beard & Björgvinsson, 2014).

### Procedure

All demographic (i.e., age / gender), clinical (i.e., primary / secondary diagnosis) and outcome data (total PHQ-9 scores / total GAD-7 scores) were extracted from an anonymised CRHTT database, held on a secure NHS server within a hospital building. All data recorded by practitioners between November 2020 and February 2021 were included in this evaluation. To maximise available data, all primary diagnoses were grouped according to ICD-10 diagnostic category. Similarly, secondary diagnoses of pervasive development disorder, hyperkinetic disorder, intellectual disability, or minor neurocognitive disorder due to acquired brain injury were combined and transformed into one binary variable (i.e., presence / absence of neurodiversity).

### Ethical Considerations

This study did not require ethical approval as it evaluated an existing NHS intervention delivered in clinical practice. According to the UK Policy Framework for Health and Social Care research, explicit participant consent is not required for service evaluations involving the retrospective use of anonymized information captured during the provision of routine clinical care. However, the study was registered and approved as a service evaluation with a local NHS trust Research and Development Department. Furthermore, all data was kept anonymised and securely in accordance with Good Clinical Practice and the Declaration of Helsinki.

### Analyses

To assess the effects of the CTB on routine outcome measures across three time points (time 1, time 2 and time 3) a series of linear trends were computed. Each time point corresponded with each CTB session number. With up to three observations per participant, two-level random effects models were fitted to the data to account for this nested structure. Linear time trends were computed as time-interval specific trends did not significantly improve the model fit (Likelihood ration tests: PHQ-9 ${\chi }_{1}^{2}=3.18, p=0.074 ;$ GAD${\chi }_{1}^{2}= 1.83, p= 0.177$). Random slope models, with a random intercept for individual and slope for time, were an overall better fit to the data than random intercept models (Likelihood ration tests: PHQ-9 ${\chi }_{1}^{2}=58.61, p<0.001 ;$ GAD${\chi }_{1}^{2}= 72.67, p< 0.001$). Therefore, linear time trends using two-level random slope models were fitted for all analyses.

## Results

### Demographics

Data from 399 participants were included in the analyses. Of these, 111 had a diagnosis of neurotic, stress-related and somatoform disorders (27.8%), 70 had mood (affective) disorders (17.5%), 37 had disorders of adult personality and behaviour (9.3%), nine had mental and behavioural disorders due to psychoactive substance use (2.3%), seven had schizophrenia, schizotypal and delusional disorders (1.8%) and one had behavioural syndromes associated with physiological disturbances and physical factors. Mental health diagnoses were unknown for 164 participants (41.1%). In terms of neurodiversity, 50 participants had a positive diagnosis (12.5%), whereas 349 participants had no recorded neurodivergence (87.5%). All baseline demographic and clinical information and summary PHQ-9 and GAD-7 scores across timepoints are presented in Table 1. Across all participants, average time-span in days between CTB sessions was comparable (Session 1 – Session 2: M = 5.43, SD = 4.04; Session 2 – Session 3: M = 5.72, SD = 3.98). The mean time between inception and completion of CTB sessions (i.e. Session 1 – Session 3) was 11.15 days (SD = 5.83).

**Table 1:** Baseline demographic and clinical information and summary PHQ-9 and GAD-7 scores over time

		Time 1
**Baseline characteristics**		
Age		
Median (IQR)		35 (25, 49)
Min, Max		18, 80
Gender		
Female		205 (51.4%)
Male		194 (48.6%)
**Mental Health Diagnosis**		
Behavioural syndromes associated with physiological disturbances and physical factors		1 (0.3%)
Disorders of adult personality and behaviour		37 (9.3%)
Mental and behavioural disorders due to psychoactive substance use		9 (2.3%)
Mood [affective] disorders		70 (17.5%)
Neurotic, stress-related and somatoform disorders		111 (27.8%)
Schizophrenia, schizotypal and delusional disorders		7 (1.8%)
Unknown		164 (41.1%)
**Neurodivergent Diagnosis**		
Yes		50 (12.5%)
No/Unknown*		349 (87.5%)
Hyperkinetic Disorders		29 (7.3%)
Intellectual Disability		6 (1.5%)
Minor neurocognitive disorder due to acquired brain injury		1 (0.3%)
Pervasive Developmental Disorders		14 (3.5%)
Not stated		349 (87.5)

		Time 1	Time 2	Time 3
**Psychological Measures**				
PHQ-9				
Median (IQR)		22 (17, 25)	20 (14, 24)	18 (12, 23)
Min, Max		1, 27	0, 27	0, 27
n		391	317	250
GAD-7				
Median (IQR)		17 (14, 19.5)	16 (11, 19)	14 (8, 18)
Min, Max		3, 22	0, 12	0, 21
n		392	317	250

### Trend Analyses

#### Do PHQ-9 and GAD-7 Scores Change over time?

Total scores on the PHQ-9 and GAD-7 showed a significantly decreasing trend over time (PHQ-9: β = − 1.6, 95% CI (− 1.9, − 1.3); GAD-7: β = − 1.5, 95% CI (− 1.8, − 1.2) (Table 2).

**Table 2:** Trends in PHQ-9 and GAD-7 scores over time

Fixed effects	PHQ-9			GAD-7		
	Estimate	95% CI	P value	Estimate	95% CI	P value
Constant	20.1	(19.5, 20.6)	< 0.001	16.0	(15.6, 16.5)	< 0.001
Time	− 1.6	(− 1.9, − 1.3)	< 0.001	− 1.5	(− 1.8, − 1.2)	< 0.001

Random effects	Estimate	95% CI		Estimate	95% CI	
Between-individual intercept variance	25.3	(21.1, 30.3)		15.4	(12.8, 18.6)	
Between-individual slope variance	2.9	(1.8, 4.6)		3.1	(2.1, 4.5)	
Between-individual intercept-slope covariance	2.7	(0.9, 4.5)		1.0	(− 0.3, 2.3)	
Within-individual variance	7.8	(6.6, 9.3)		5.2	(4.4, 6.2)	

#### Are Trends in PHQ-9 and GAD-7 Scores Similar for men and Women?

For women, total scores on the PHQ-9 and GAD-7 showed a significantly decreasing trend over time (PHQ-9: β= − 1.6, 95% CI (− 2.0, − 1.1); GAD-7: β= − 1.4, 95% CI (− 1.8, − 1.0)). For men, total PHQ-9 and GAD-7 scores showed comparably decreasing and significant trends over time (PHQ-9: β= -1.6, 95% CI (− 2.1, − 1.2); GAD-7: β= − 1.6, 95% CI (− 2.1, − 1.2) (Table 3).

**Table 3:** Trends in PHQ-9 and GAD-7 scores over time according to gender

Fixed effects	PHQ-9			GAD-7		
	Estimate	95% CI	P value	Estimate	95% CI	P value
Constant	20.8	(20.0, 21.6)	< 0.001	16.5	(15.9, 17.1)	< 0.001
Time	− 1.6	(− 2.0, -1.1)	< 0.001	− 1.4	(− 1.8, − 1.0)	< 0.001
Gender*Time	− 0.1	(− 0.7, 0.6)	0.835	− 0.2	(− 0.7, 0.4)	0.584

Random effects	Estimate	95% CI		Estimate	95% CI	
Between-individual intercept variance	24.7	(20.6, 29.7)		15.1	(12.5, 18.2)	
Between-individual slope variance	2.9	(1.8, 4.6)		3.1	(2.1, 4.4)	
Between-individual intercept-slope covariance	2.7	(0.9, 4.5)		0.9	(− 3.6, 2.5)	
Within-individual variance	7.9	(6.7, 9.3)		5.2	(4.4, 6.2)	

#### Are Trends in PHQ-9 and GAD-7 Scores Similar Across ages?

Whilst there was no significant interaction between age and time in PHQ-9 scores, GAD-7 scores in older participants showed a significantly steeper decreasing trend over time compared with younger participants: for every additional 10 years the trend decreased by 0.2 (95% CI (− 0.04, − 0.0), *p* = 0.042) (Table 4).

**Table 4:** Trends in PHQ-9 and GAD-7 scores over time according to age

Fixed effects	PHQ-9			GAD-7		
	Estimate	95% CI	P value	Estimate	95% CI	P value
Constant	21.3	(19.8, 22.9)	< 0.001	16.7	(15.5, 17.9)	< 0.001
Time	− 0.9	(− 1.8, -0.1)	0.033	− 0.7	(− 1.5, 0.0)	0.058
Age*Time	− 0.2	(− 0.4, 0.0)	0.102	− 0.2	(− 0.4, − 0.0)	0.042

Random effects	Estimate	95% CI		Estimate	95% CI	
Between-individual intercept variance	25.0	(20.8, 30.0)		15.3	(12.7, 18.5)	
Between-individual slope variance	2.8	(1.8, 4.6)		3.0	(2.1, 4.4)	
Between-individual intercept-slope covariance	2.6	(0.8, 4.4)		0.9	(− 0.4, 2.2)	
Within-individual variance	7.9	(6.7, 9.3)		5.2	(4.3, 6.1)	

#### Are Trends in PHQ-9 and GAD-7 Scores Similar Across Diagnoses?

Both PHQ-9 and GAD-7 total scores showed a significantly decreasing trend over time in participants with a diagnosis of mood (affective) disorders (PHQ-9: β= − 1.7, 95% CI (− 2.5, − 1.0); GAD-7: β= − 1.6, 95% CI (− 2.3, − 1.0)) and neurotic, stress-related and somatoform disorders (PHQ-9: β= − 1.5, 95% CI (− 2.1, − 0.99); GAD-7: β= − 1.8, 95% CI (− 2.3, − 1.2)). For participants with a diagnosis of behavioural syndromes associated with physiological disturbances and physical factors, GAD-7 total scores showed a strong, significantly decreasing trend over time (β= − 5.0, 95% CI (− 9.7, − 0.3). All other diagnoses showed decreasing, albeit non-significant, trends in PHQ-9 and GAD-7 total scores over time, except for GAD-7 scores in those with a diagnosis of schizophrenia, schizotypal and delusional disorders, which increased, albeit non-significantly (Table 5).

**Table 5:** Trends in PHQ-9 and GAD-7 scores over time according to mental health diagnosis

MH diagnosis	PHQ-9			GAD-7		
	Estimate	95% CI	P value	Estimate	95% CI	P value
0	− 5.0	(− 10.3, 0.3)	0.065	− 5.0	(− 9.7, − 0.3)	0.038
1	− 0.8	(− 1.9, 0.3)	0.160	− 0.4	(− 1.4, 0.6)	0.460
2	− 1.2	(− 3.5, 1.2)	0.327	− 1.1	(− 3.2, 0.9)	0.273
3	− 1.7	(− 2.5, − 1.0)	0.000	− 1.6	(− 2.3, − 1.0)	0.000
4	− 1.5	(− 2.1, − 0.9)	0.000	− 1.8	(− 2.3, − 1.2)	0.000
5	− 0.1	(− 2.2, 2.0)	0.895	0.8	(− 1.1, 2.6)	0.421

0 - Behavioural syndromes associated with physiological disturbances and physical factors, 1 - Disorders of adult personality and behaviour, 2 – Mental and Behavioural disorders due to psychoactive substance use, 3 - Mood [affective] disorders, 4 - Neurotic, stress-related and somatoform disorders, 5 - Schizophrenia, schizotypal and delusional disorders

#### Are Trends in PHQ-9 and GAD-7 Scores Similar Across the Spectrum of Neurodiversity?

For participants classified as ‘neurotypical’, PHQ-9 and GAD-7 total scores showed a significantly decreasing trend over time (PHQ-9: β = − 1.8, 95% CI (− 2.1, − 1.4); GAD-7: β = − 1.6, 95% CI (− 1.9, − 1.3)). Although there were no significant differences in GAD-7 scores between those classified as ‘neurotypical’ and ‘neurodiverse’, PHQ-9 scores for participants classified as ‘neurodiverse’ showed a significantly less reducing trend over time (Table 6).

**Table 6:** Trends in PHQ-9 and GAD-7 scores over time according to presence / absence of neurodiversity

Fixed effects	PHQ-9			GAD-7		
	Estimate	95% CI	P value	Estimate	95% CI	P value
Constant	20.0	(19.4, 20.6)	< 0.001	16.0	(15.5, 16.4)	< 0.001
Time	− 1.8	(− 2.1, -1.4)	< 0.001	− 1.6	(− 1.9, − 1.3)	< 0.001
Neuro*Time	1.2	(0.3, 2.1)	0.009	0.8	(− 0.04, 1.6)	0.062

Random effects	Estimate	95% CI		Estimate	95% CI	
Between-individual intercept variance	25.1	(20.9, 30.2)		15.4	(12.7, 18.5)	
Between-individual slope variance	2.7	(1.7, 4.5)		3.0	(2.1, 4.4)	
Between-individual intercept-slope covariance	2.6	(0.8, 4.4)		0.9	(− 0.4, 2.2)	
Within-individual variance	7.9	(6.7, 9.3)		5.2	(4.4, 6.2)	

## Discussion

This retrospective service evaluation aimed to examine the clinical effects of the CTB, a brief, skills-based intervention delivered in one CRHTT in the UK. Trends in PHQ-9 and GAD-7 scores were analysed across three time points in a sample of 399 participants who received the CTB from November 2020 to February 2021. Differential trends according to age, gender, mental health diagnosis and neurodiversity were also evaluated.

Our results demonstrated significantly decreasing trends in PHQ-9 and GAD-7 scores across time in participants who accessed the CTB. The magnitude of these trends was similar for depression and anxiety and for both men and women. Although there were no significant differences in PHQ-9 trends according to age, comparative to their younger counterparts, older participants had a significantly greater reduction of GAD-7 scores across time. This is somewhat surprising, given evidence that psychological interventions for anxiety appear less effective for older people than for adults of working age (Gould et al., 2012). However, there is evidence that older people can benefit significantly from brief interventions delivered via other tele-health platforms (Titov et al., 2016), which may overcome some of the obstacles they face accessing specialised mental health care (Mozer et al., 2008).

PHQ-9 and GAD-7 total scores decreased over time for participants with a diagnosis of mood disorder or neurotic, stress-related and somatoform disorders. A much greater reduction was observed in people with a diagnosis of behavioural syndromes associated with physiological disturbances and physical factors; however, this effect was only statistically significant for anxiety. For people with all other mental health diagnoses, there were very little changes to anxiety or depression scores over time. It is noteworthy that the small number of participants included with a diagnosis of behavioural syndromes, mental or behavioural disorders due to psychoactive substance use, and schizophrenia, schizotypal and delusional disorders may render these findings invalid. Therefore, further research is needed to ascertain if the CTB requires adaptation for these groups. However, the point estimates suggest the CTB may hold promise as a transdiagnostic intervention for those presenting in crisis. Moreover, whereas trends in PHQ-9 and GAD-7 scores significantly decreased for ‘neurotypical’ participants, depression for those classified as ‘neurodiverse’ was relatively constant over time. Whether the CTB in its current form is sensitive to the needs of those with neurodiversity remains unclear.

Overall, the significant trends reported in this study suggest the CTB might be helpful in reducing anxiety and depression in people accessing CRHTT settings. These findings align with the outcomes of brief interventions in the treatment of suicidal thoughts and / or behaviours delivered across other urgent care settings (Yardley et al., 2019; Guthrie et al., 2001; McCabe et al., 2018; Chopra et al., 2021). They also lend support to the growing literature on the utility of telephonic interventions in the treatment of mental health problems in primary (Simon et al., 2004) and acute care (Bidargaddi et al., 2015) and suggest these may hold similar promise in CRHTT settings. The CTB was designed as a transdiagnostic, skills-based intervention in response to COVID-19, the need for flexible methods of service delivery (Zhou et al., 2020) and to overcome the difficulties with access to psychological interventions in CRHTT settings (Ebrahim, 2021). The CTB is acceptable, appears to have a clinical impact on anxiety and depression, and does not require specialist training to deliver. Therefore, it could represent an important pathway in CRHTT service provision, maximising the reach of psychological interventions for those experiencing crises whilst establishing and embedding direct and indirect roles for clinical psychologists across urgent care (ACP, 2021). It might also contribute to positive CRHTT outcomes (Crompton & Daniel, 2007).

This service evaluation has several limitations. Firstly, as there was no comparator group, only cautious inferences can be made for any treatment effect. Changes in PHQ-9 and GAD-7 scores could be due to several factors including natural variation, regression to the mean, situational factors, the Hawthorne effect, treatment as usual (including the effect of crisis safety planning), or the psychological components of frequent contacts (McCabe et al., 2018). Although it is likely the CTB has *some* effect, especially considering its high acceptability (Mulligan et al., 2022), more rigorous studies employing control groups and trial methodology are required to determine this. Secondly, the retrospective nature of this evaluation meant that demographic, clinical and outcome variables were restricted to those previously recorded. Research evaluating trends according to race, ethnicity, disability, and sexual orientation; eliciting qualitative feedback, or recording unintended side effects / incidents attributable to the CTB are required to supplement the trends found and clarify its safety and tolerability. Thirdly, although composite measures of ‘neurodiversity’ and ICD-10 diagnostic categories were formed to maximise available data, missing data was still problematic, and the validity of these variables is questionable. Fourthly, as the CTB was delivered in routine clinical practice, there were no direct analyses of treatment fidelity. Nevertheless, all practitioners were closely supervised by one clinical psychologist who maintained oversight throughout the study. Finally, the current results are limited by the absence of longer-term follow-up, as this fell outside of the remit of routine CTB provision and would have required ethical approval. Future research should consider implementing follow-up assessments to evaluate durability in outcomes.

## Conclusion

The significant results reported in this study suggest the CTB could be helpful in reducing anxiety and depression for people experiencing crises; although the magnitude of trends appear to vary according to age, diagnosis, and neurodiversity. The CTB could be one solution to the nationwide difficulties faced by service users in terms of access to acceptable and effective psychological interventions in CRHTT settings (Ebrahim, 2021). However, randomised controlled pilot trials .are now required to explicitly test the acceptability, feasibility, and clinical effects of the CTB, to expand on these initial results and inform its future delivery.
